# Mechanism of cardioprotective effect of erythropoietin-induced preconditioning in rat heart

**DOI:** 10.4103/0253-7613.68421

**Published:** 2010-08

**Authors:** Kavita Garg, Harlokesh N. Yadav, Manjeet Singh, P. L. Sharma

**Affiliations:** Department of Pharmacology, I.S.F College of Pharmacy, Moga - 142 001, Punjab, India

**Keywords:** Epo preconditioning, ischemic preconditioning, JAK-2, PI-3K, PKC

## Abstract

**Objective:**

The cardioprotective potential of human recombinant erythropoietin (alpha) (Epo) against ischemia-reperfusion-induced injury is well known. But, the underlying mechanisms are not well elucidated. The aim of this study was to characterize the mechanism involved in the cardioprotective effect of Epo-induced preconditioning in isolated rat heart.

**Materials and Methods:**

The heart was mounted on a Langendorff apparatus. After 10 min of stabilization, four cycles of ischemic preconditioning (IPC) were given followed by 30 min of global ischemia and 120 min of reperfusion. Epo preconditioning was induced by four cycles of 5-min perfusion of K-H solution containing Epo (1.0 U/ml) followed by 5 min perfusion with K-H solution. Myocardial infarct size was estimated macroscopically using the triphenyltetrazolium chloride staining technique. The extent of myocardial injury was measured by release of lactate dehydrogenase and creatine kinase-MB in the coronary effluent.

**Results:**

The present study demonstrates that Epo preconditioning was almost as effective as IPC. Administration of Wortmannin (100 nM), a PI-3K inhibitor, or Chelerythrine (1 µM), a protein kinase-C (PKC) inhibitor, or AG490 (5 µM), a JAK-2 inhibitor, significantly attenuated the cardioprotective effects of Epo-induced preconditioning.

**Conclusion:**

Our result suggest that the cardioprotective potential of Epo-induced preconditioning in isolated rat heart was due to an interplay of the JAK-2, PI-3K and PKC pathways. Inhibition of any one of the three pathways was sufficient to block the cardioprotective effect of Epo-induced preconditioning in isolated rat heart.

## Introduction

Ischemic preconditioning (IPC) is an endogenous protective phenomenon in which brief episodes of sublethal ischemia followed by reperfusion increase the resistance of the myocardium to subsequent sustained ischemia of longer duration.[1] It has been reported that IPC produces cardioprotection by the activation of Janus kinase/signal transducer and activator of transcription (JAK/STAT),[2,3] phosphatidylinositol-3-kinase (PI-3Kinase)[4] and protein kinase-C (PKC).[5] Erythropoietin (Epo) is a glycoproteinious hormone of molecular weight 30 kDa, produced by the kidney, which regulates the proliferation, differentiation and maturation of erythrocytes.[6] Its plasma levels increase during hypoxia through hypoxia-inducible factor-1 (HIF-1).[7] Moreover, it is also produced by the brain, liver, uterus, trophoblasts, astrocytes and macrophage of the bone marrow.[8] Apart from erythropoiesis, Epo enhances the activity of stem cells,[9] reverses vasospasm,[10] also used in the treatment of chronic renal failure,[11] protects the brain against ischemic injury[12] and preserves the integrity of the endothelium.[13] It has been reported that pretreatment with Epo produces cardioprotection against I/R-induced injury by JAK/STAT,[14] PI-3K/Akt[15] and PKC[16] signaling pathways.

It has been reported that both IPC and Epo produce cardioprotection through common signaling pathways: PI-3K, PKC and JAK/STAT.[4,5,15–22] Glycogen synthase kinase-3β (GSK-3β) is a downstream common pathway of the PI-3K, PKC and JAK/STAT[23] pathways and its phosphorylation is noted to produce cardioprotection.[24,25] However, Nishihara *et al*.[26] reported that administration of Epo with IPC exerts additional cardioprotection, mediated through enhanced phosphorylation of GSK-3β, and suggested that the Epo-mediated primary signaling pathway is different from that of the IPC-induced signaling cascade. Thus, the objective of this study was to determine the relative role of Epo-induced cell survival signaling pathways, including PI-3K, PKC and JAK/STAT, in isolated rat heart.

## Materials and Methods

The experimental protocol used in the present study was approved by the Institutional Animal Ethics Committee.

### Drugs and chemicals:

Human recombinant Epo (alpha) (Gennova Biopharmaceuticals Ltd., Pune, India), Wortmannin, chelerythrine and AG490 (Sigma Aldrich [P] Ltd., Bangalore, India) were dissolved in dimethyl sulfoxide (DMSO) and the final concentration of DMSO in K-H solution was 0.02%. All other reagents used in this study were of analytical grade and were always freshly prepared before use.

### Isolated rat heart preparation:

Rats were administered heparin (500 IU/L, i.p.) 20 min before their sacrifice by cervical dislocation. The heart was rapidly excised and immediately mounted on Langendorff’s apparatus[27] and was retrogradely perfused at a constant pressure of 80 mmHg with Kreb’s Henseleit (KH) buffer (NaCl 118 mM; KCl 4.7 mM; CaCl_2_ 2.5 mM; MgSO_4_.7H_2_O 1.2 mM; KH_2_PO_4_ 1.2 mM; C_6_H_12_O_6_ 11 mM), pH 7.4 and bubbled with 95% O_2_ and 5% CO_2_. The flow rate was maintained at 7–9 ml/min using a Hoffman’s screw. The heart was enclosed in a double-wall jacket, the temperature of which was maintained by circulating water heated at 37°C. IPC was produced by closing the inflow of K-H solution for 5 min followed by 5 min of reperfusion. Four such episodes were employed. Global ischemia was produced for 30 min followed by 120 min of reperfusion. The coronary effluent was collected before ischemia, immediately, 5 min and 30 min after reperfusion for estimation of lactate dehydrogenase (LDH) and creatine kinase-MB (CK-MB).

### Erythropoietin-induced preconditioning:

Four cycles, each cycle consisting of 5-min perfusion of K-H solution containing Epo (1.0 U/ml) followed by 5 min perfusion with K-H solution (free of Epo), and followed by 30 min global ischemia and 120 min reperfusion were used.

### Assessment of myocardial injury:

The myocardial infarct size was measured using the triphenyltetrazolium chloride (TTC) staining method while the level of LDH (Siemens Medical Solution Diagnostics Ltd., Baroda, India) and CK-MB (Nicholas Piramal India Ltd., Mumbai, India) in coronary effluents was estimated using commercially available kits. Values of LDH and CK-MB were expressed in international units per liter (IU/L).

### Assessment of myocardial infarct size:

The heart was removed from the Langendorff’s apparatus. Both the atria and the root of the aorta were excised and the ventricles were kept overnight at a temperature of -4°C. Frozen ventricles were sliced into uniform sections of about 1–2 mm thickness. The slices were incubated in 1% w/v TTC at 37°C in 0.2M tris-chloride buffer for 30 min. The normal myocardium was stained brick red while the infarcted portion remained unstained. Infarct size was measured by the volume method.[28]

### Experimental protocol:

**Figure 1:**
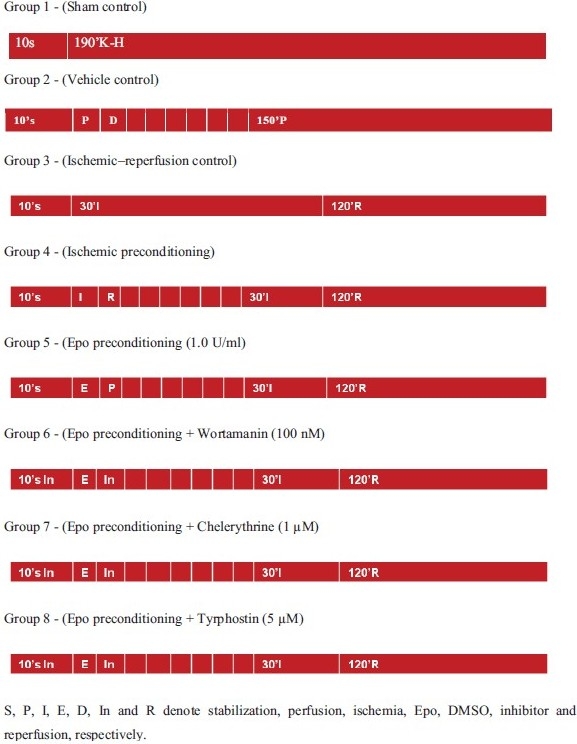
Diagrammatic representation of the experimental protocol

A diagrammatic representation of the experimental protocol is shown in Figure 1.

In all groups, isolated rat heart was perfused with K-H solution and allowed to stabilize for 10 min. Group 1: (Sham control; *n* = 6) After stabilization, the heart was perfused continuously with K-H buffer for 190 min without subjecting it to global ischemia. Group 2: (Vehicle control: *n* = 6) After stabilization, the heart was perfused for 5 min with K-H containing DMSO (0.02%) followed by 5 min perfusion with K-H solution free of DMSO; four such cycles were repeated. Then, the preparation was subjected to 150 min of perfusion. Group 3: (Ischemia-reperfusion control; *n* = 6) After stabilization, the heart was subjected to 30 min of global ischemia followed by 120 min of reperfusion. Group 4: (IPC control; *n* = 6) Four cycles of IPC were given just after stabilization. Each cycle comprised of 5 min ischemia and 5 min reperfusion with K-H solution and then followed by 30 min global ischemia and 120 min reperfusion. Group 5: (Epo preconditioning: *n* = 6) Just after stabilization, the heart was subjected to four cycles of Epo preconditioning, each cycle comprising of 5 min perfusion with K-H solution containing Epo (1.0 U/ml) followed by 5 min perfusion with K-H solution free of Epo and further followed by 30 min of global ischemia and 120 min of reperfusion. Group 6: (Epo preconditioning in Wortmannin-perfused rat heart; *n* = 6) During 10 min of stabilization, the heart was perfused with K-H solution containing 100 nM of Wortmannin followed by four repeated episodes of 5 min perfusion of K-H solution containing Epo (1.0 U/ml) followed by 5 min perfusion with K-H solution containing (100 nM) Wortmannin. Then, the preparation was subjected to 30 min global ischemia followed by 120 min reperfusion. Group 7: (Epo preconditioning in Chelerythrine-perfused rat heart; *n* = 6) During stabilization, the heart was perfused with K-H solution containing 1 µM Chelerythrine followed by four repeated episodes of 5 min perfusion of K-H solution containing Epo (1.0 U/ml) followed by 5 min perfusion with K-H solution containing (1 µM) Chelerythrine followed by 30 min global ischemia and 120 min reperfusion. Group 8: (Epo preconditioning in AG490-perfused rat heart; *n* = 6) The heart was perfused with K-H solution containing 5 µM AG490 during the stabilization followed by four repeated episodes of 5 min perfusion of K-H solution containing Epo (1.0 U/ml) followed by 5 min perfusion with K-H solution containing (5 µM) AG490 further followed by 30 min of global ischemia and 120 min of reperfusion.

### Statistical Analysis

All values were expressed as mean ± standard deviation (SD). Statistical analysis was performed using Sigmastat Software. The values were statistically analysed using one-way analysis of variance (ANOVA) followed by Tukey’s multiple comparison test. Value of *P* <0.05 was considered as statistically significant.

## Results

**Figure 2:**
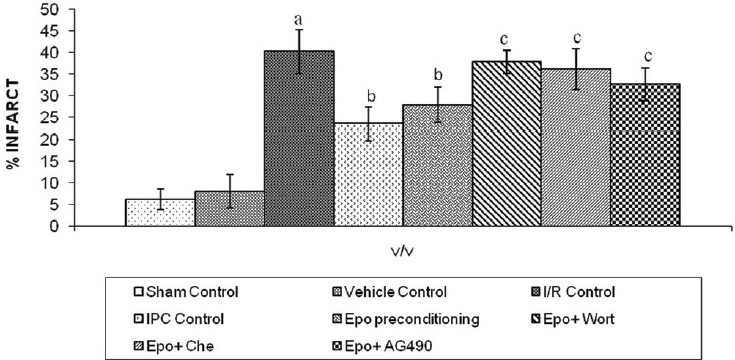
Effect of I/R, IPC and Epo preconditioning and effect of Wortmanin, Chelerythrine and AG490 on myocardial infarct size I/R, IPC, Epo, Wort, Che and AG490 denote ischemia–reperfusion, ischemic preconditioning, erythropoietin, Wortmannin, Chelerythrine and AG 490, respectively. Values are expressed as mean ± SD, a = *P <*0.05 vs. sham control; b = *P <*0.05 vs. I/R control; c = *P <*0.05 vs. Epo preconditioning. ANOVA followed by Tukey’s multiple comparison test

Effect of IPC, Epo preconditioning and pharmacological interventions on myocardial infarct size:

Both IPC and Epo preconditioning significantly attenuated ischemia–reperfusion-induced increase in myocardial infarct size. Administration of Wortmannin or Chelerythrine or AG490 significantly (*P* < 0.05) reversed the Epo preconditioning-induced decrease in myocardial infarct size [Figure 2]. The difference in the inter-se efficacy of the three inhibitors were not statistically significant (*P* > 0.05).

### Effect of IPC, Epo preconditioning and pharmacological interventions on release of LDH

**Figure 3:**
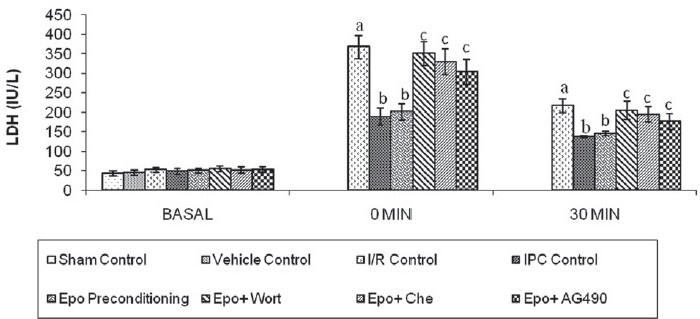
Effect of I/R, IPC and Epo preconditioning and effect of Wortmanin, Chelerythrine and AG490 on the release of lactate dehydrogenase. I/R, IPC, Epo, Wort, Che and AG490 denote ischemia–reperfusion, ischemic preconditioning, erythropoietin, Wortmannin, Chelerythrine and AG 490, respectively. Values are expressed as mean ± SD; a = *P <*0.05 vs. Sham control; b = *P <*0.05 vs. I/R control; c = *P <*0.05 vs. Epo preconditioning. ANOVA followed by Tukey’s multiple comparison test

Both IPC and Epo preconditioning significantly attenuated ischemia-reperfusion-induced increase in release of LDH in the coronary effluent. Administration of Wortmannin or Chelerythrine or AG490 significantly (*P* < 0.05) reversed the Epo preconditioning-induced decrease in the release of LDH in the coronary effluent [Figure 3]. The difference in the inter-se efficacy of the three inhibitors was not statistically significant (*P* > 0.05).

### Effect of IPC, Epo preconditioning and pharmacological interventions on release of CK-MB

**Figure 4:**
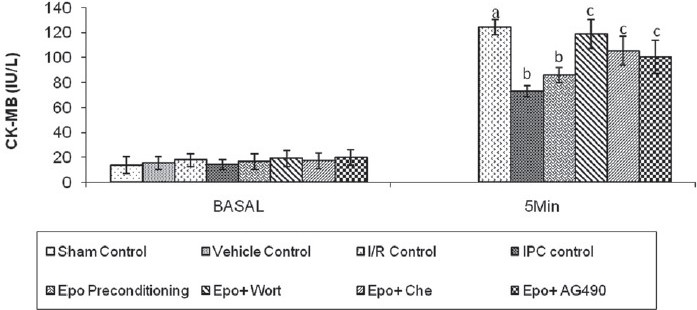
Effect of I/R, IPC and Epo preconditioning and effect of Wortmanin, Chelerythrine and AG490 on the release of creatine kinase-MB. I/R, IPC, Epo, Wort, Che and AG490 denote ischemia–reperfusion, ischemic preconditioning, erythropoietin, Wortmannin, Chelerythrine and AG 490, respectively. Values are expressed as mean ± SD; a = *P <*0.05 vs. Sham control; b = *P <*0.05 vs. I/R control; c = *P <*0.05 vs. Epo preconditioning. ANOVA followed by Tukey’s multiple comparison test

Both IPC and Epo preconditioning significantly attenuated ischemia-reperfusion-induced increase in release of CK-MB in the coronary effluent. Administration of Wortmannin or Chelerythrine or AG490 significantly (*P* < 0.05) reversed the Epo preconditioning-induced decrease in the release of CK-MB in the coronary effluent [Figure 4]. The difference in the inter-se efficacy of the three inhibitors was not statistically significant (*P* > 0.05).

## Discussion

Both pharmacological preconditioning and IPC remain the validated approaches to minimize the ischemia-reperfusion-induced damage of the myocardium. Also, the cardioprotective effect of Epo has been well documented in different animal models, including isolated cardiomyocytes,[29] isolated Langendorff heart preparation[30] and coronary artery ligation models.[31] The reported molecular mechanism of Epo-induced cardioprotection is believed to be almost identical to that of IPC-induced cardioprotection.

IPC produces cardioprotection by phosphorylation and inhibition of GSK-3β.[32] It has been reported that JAK/STAT, PI-3K and PKC are the common upstream kinases of GSK-3β[23] and that IPC produces cardioprotection by activation of the JAK/STAT,[2,3] PI-3K[4] and PKC[5] pathways. In the present study, four cycles of Epo preconditioning produced cardioprotection, measured in terms of I/R-induced decrease in infarct size and decrease in the release of LDH and CK-MB. Moreover, it has been documented that early perfusion of Epo protects the ischemia-reperfusion-induced injury by JAK/STAT,[33] PI-3K[34] and PKC.[16] Hence, it may be suggested that the observed Epo preconditioning-induced cardioprotection may be mediated through the same pathways.

Recently, Nishihara *et al*.[26] reported that administration of Epo with IPC produces additional cardioprotection in the rat heart against I/R-induced injury by enhancing the phosphorylation of GSK-3β and also that the relative importance of PKC and PI-3K pathways differs in the Epo-induced and IPC-induced cardioprotection against ischemia-reperfusion injury. It may be that cardioprotective stimuli of Epo-induced preconditioning may not be parallel to stimuli induced by IPC. GSK-3β is a unique downstream target[23] of various signaling pathways, viz. PI-3K, PKC and JAK/STAT,[4,5,15–22] observed during IPC- and Epo-induced cardioprotection. In our study, pretreatment with Wortmannin (PI-3K inhibitor) or Chelerythrine (PKC inhibitor) or AG490 (JAK2 inhibitor) significantly and almost equally attenuated the Epo preconditioning-induced decrease in myocardial infarct size and release of LDH and CK-MB in the coronary effluent, indicating that Epo simultaneously activates the JAK/STAT, PI-3K and PKC pathways, which play an important role in Epo-preconditioning-induced cardioprotection. There was no statistically significant differences in the inter-se efficacy of the three inhibitors used. Thus, we found no relative difference in the role of PKC, PI-3K and JAK2 in Epo-induced cardioprotection. This difference between our results and those of Nishihara *et al*.[26] may be due to the difference in the experimental models used. The activation of any one of these pathways is sufficient to inactivate the common downstream GSK-3β[32] that protects the cardiac myocytes from ischemia-reperfusion injury in rat hearts.

Based on the results of the present study, it may be concluded that Epo-induced preconditioning was almost as effective as IPC (*P* > 0.05) and EPO preconditioning-induced cardioprotection in the isolated rat heart is mediated through an interplay of the JAK-2, PI-3K and PKC pathways. The order of effectiveness for attenuation of erythropoietin preconditioning was Wort>Che>AG490 in the dosages used, but these inter-se differences in the efficacy were not statistically significant.
